# Case Report: Post-Total anomalous pulmonary venous connection pulmonary hypertension - novel treatment using sirolimus and atrial flow regulator implantation

**DOI:** 10.3389/fcvm.2026.1783773

**Published:** 2026-05-13

**Authors:** Stasa Krasic, Antony Hermuzi, Ivan Dizdarevic, Vesna Topic, Nevena Djorovic, Mihail Basa, Vladislav Vukomanovic

**Affiliations:** 1 Cardiology Department, Mother and Child Health Institute of Serbia, Belgrade, Serbia; 2 Faculty of Medicine, University of Belgrade, Belgrade, Serbia; 3 Adult Congenital and Paediatric Heart Unit, Freeman Hospital, Newcastle upon Tyne Hospitals NHS Foundation Trust, Newcastle upon Tyne, United Kingdom; 4 Cardiac Surgery Department, Mother and Child Health Institute of Serbia, Belgrade, Serbia; 5 Radiology Department, Mother and Child Health Institute of Serbia, Belgrade, Serbia; 6 Pulmology Department, Mother and Child Health Institute of Serbia, Belgrade, Serbia

**Keywords:** atrial flow regulator (AFR) - device, down sizing, pulmonary hypertension, sirolimous, total anomalous of pulmonary venous connection

## Abstract

**Objective:**

In patients who have undergone surgical repair of TAPVC and who develop treatment-resistant pulmonary vein stenosis and postcapillary pulmonary hypertension, pre-capillary pulmonary hypertension may also develop as a consequence of arterial remodelling. We present a 3-year-old boy with surgically corrected TAPVC in whom AFR 8   ×   5 was implanted using a 9F delivery system due to end-stage precapillary PH.

**Case report:**

In a male term newborn, obstructive supracardiac TAPVR was diagnosed, and he underwent surgical repair on the third day of life. At three months of age, he was hospitalised for respiratory distress, feeding intolerance, and early fatigability. He underwent re-operation using a sutureless repair technique with creation of an atrial septal defect (ASD). The postoperative course was complicated by left upper pulmonary venous (LUPV) obstruction, and multiple balloon venoplasties were performed. Hemodynamic and oximetric measurements were consistent with mixed PH. Sirolimus, losartan and bosentan therapy were initiated. Systemic infections complicated the in-hospital course. Due to prolonged dependence on invasive mechanical ventilation, tracheostomy was performed. Invasive haemodynamic assessment in the 3rd year of life revealed severe precapillary PH (mean PA pressure 98 mmHg, PVR 12. 8 Wu/m2, wedge 14 mmHg). At the same time, CT angiography showed no pulmonary vein obstructions and anastomosis between the LUPV and LLPV. A month after catheterisation, he was admitted to the ICU due to deterioration of his general condition and progression of PH (body weight 10 kg). Inhaled NO, along with high-dose milrinone, was initiated; despite a favourable clinical course, we decided to proceed with implantation of an atrial flow regulator (AFR). The intervention was performed on iNO and milrinone. ASD dilatation was performed using a Z-Med 2 balloon (10   ×   20 mm). During balloon insufflation, severe bradycardia with electromechanical dissociation was observed. Adrenaline infusion was initiated, and repeated boluses were administered. AFR 8   ×   5 was implanted via 9F delivery sheath. Echocardiography showed right ventricular unloading, reduced tricuspid regurgitation, correct device placement, and a right-to-left shunt. Hemodynamic stability was rapidly achieved, allowing discontinuation of inotropes and iloprost after six days. The patient was transitioned to home mechanical ventilation, with clinical conditions gradually improving and cyclic intravenous iloprost was initiated.

**Conclusion:**

The development of precapillary PH is a serious complication following TAPVR operation. The use of a pulmonary vasodilator and AFR implantation can facilitate RV unloading. The feasibility and clinical impact of AFR implantation via an undersized delivery system in paediatric end-stage PH are promising as a bridge to transplant.

## Introduction

The reported incidence and prevalence of pulmonary hypertension (PH) related to congenital heart disease (CHD) are 2.2 and 15.6 cases per million, respectively (1). Group 1 PH encompasses PH linked with CHD, including conditions with a pretricuspid shunt such as total anomalous pulmonary venous connection (TAPVC), and PH that persists or recurs following surgical repair of CHD. Post-surgical PH in CHD patients is associated with a poor prognosis (1).

In patients who have undergone surgical correction of TAPVC, especially obstructive TAPVC, ongoing or recurrent precapillary pulmonary hypertension (PH) remains a major risk for morbidity and mortality (2). This condition arises from pulmonary vascular remodelling and elevated pulmonary vascular resistance (PVR), which may not fully resolve after the initial surgery. Before surgical correction, a left-to-right shunt causes volume and pressure overload of the right heart chambers and results in an underdeveloped or “underfilled” left-sided heart chamber. This chronic state can lead to pathological remodelling, including thickening of the medial layer of the pulmonary arteries and muscle extension into distal arterioles, thereby increasing PVR. After successful surgical repair, although abnormal blood flow is corrected, PVR may not normalise. The main risk factors include preoperative pulmonary venous obstruction, younger age, lower body weight at surgery, associated cardiac lesions, pre-existing pulmonary vascular changes, and postoperative pulmonary venous obstruction (2, 3).

Postoperative pulmonary venous obstruction is a serious complication (5%–18% incidence) caused by anastomotic narrowing, tissue reaction, or pre-existing small pulmonary veins. Management includes balloon venoplasty, percutaneous stenting, reoperation (including sutureless techniques), and, in some cases, medications such as sirolimus (4, 5).

For precapillary PH, pharmacotherapy is typically employed, with hemodynamic studies showing elevated PVR, usually performed before treatment initiation. In end-stage PH, creating a right-to-left shunt can reduce pulmonary pressure, although it causes systemic desaturation. AFR (Atrial Flow Regulator) implantation establishes a controlled atrial shunt, thereby significantly alleviating symptoms by decompressing the right heart, supporting transplant stability. A notable challenge for pediatric AFR implantation is the requirement of a 12F delivery sheath for an 8 mm device and a 14F sheath for a 10 mm device (6).

We present a 3-year-old boy with surgically corrected TAPVC in whom AFR 8  ×  5 was implanted using a 9F delivery system due to end-stage precapillary PH.

## Case report

**Figure 1:**
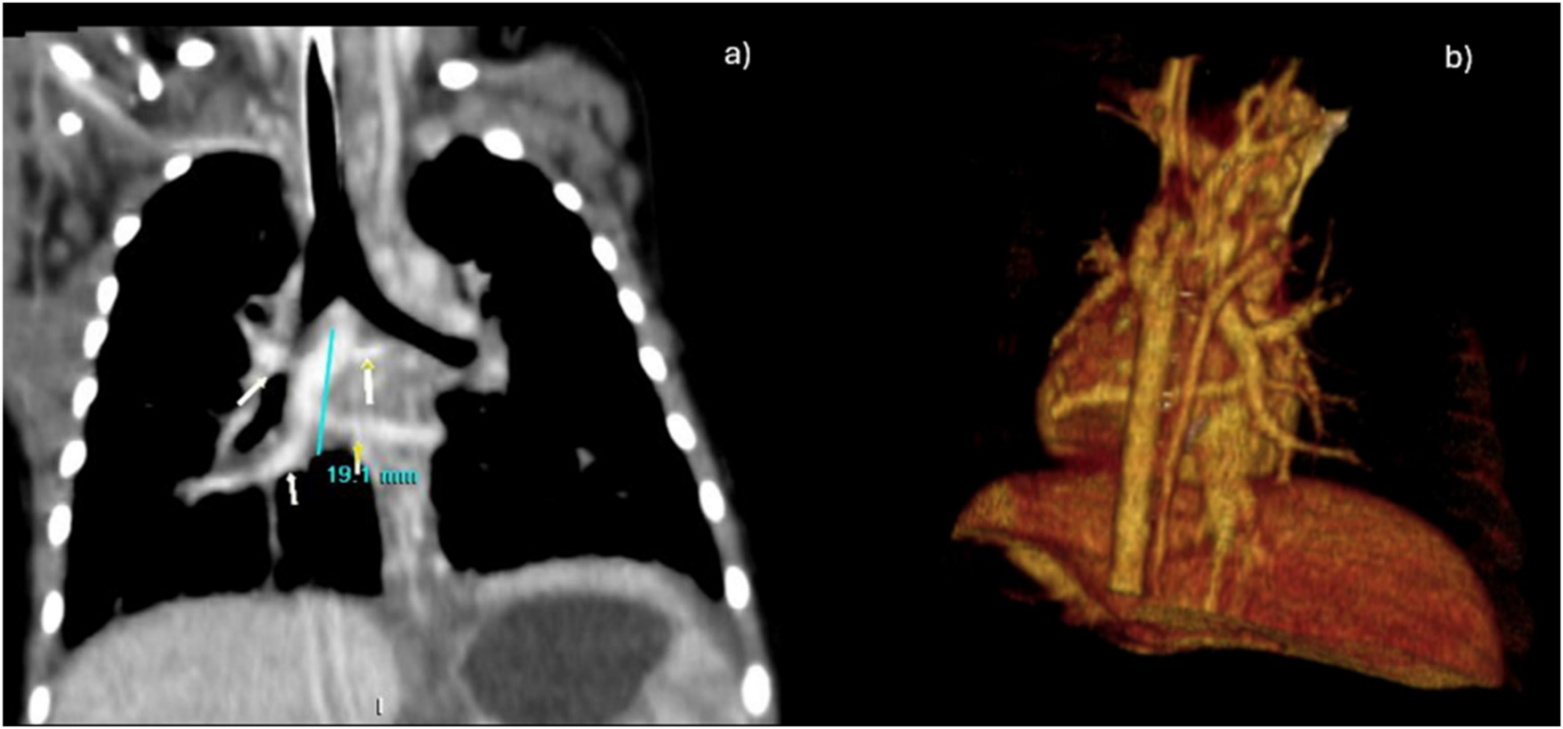
Panel **(a)** shows a grayscale coronal CT scan of the thorax with white and yellow arrows indicating anatomical landmarks of the pulmonary veins, and a blue measurement line labelled almost 20 mm (blue line) distance between the upper and lower veins. Panel **(b)** displays a 3D volume-rendered colour reconstruction of the same region, highlighting the thin vertical vein (diameter 4 mm), located right paravertebrally, into which the pulmonary veins drained.

In a male term newborn [body weight (BW) 3.3 kg], obstructive supracardiac TAPVR was diagnosed, and he underwent conventional surgical repair on the third day of life. CT angiography demonstrated anatomically unfavourable morphology and localisation of the pulmonary venous confluence, which drained into the proximal segment of the superior caval vein (SVC). The confluence was formed as a thin vertical vein (diameter 4 mm), located right paravertebrally, into which the pulmonary veins drained, with an approximate distance of 20 mm between the upper and lower veins (Figure 1). In addition, the left-sided pulmonary veins had a long prevertebral course and were compressed by surrounding structures. Furthermore, CT signs of pulmonary hypertension were present, with septal thickening and consolidation of the pulmonary parenchyma. Intracranial haemorrhage complicated the postoperative course. Echocardiography on discharge revealed the existence of the tricuspid peak pressure gradient (PG) of 37 mmHg.

**Figure 2:**
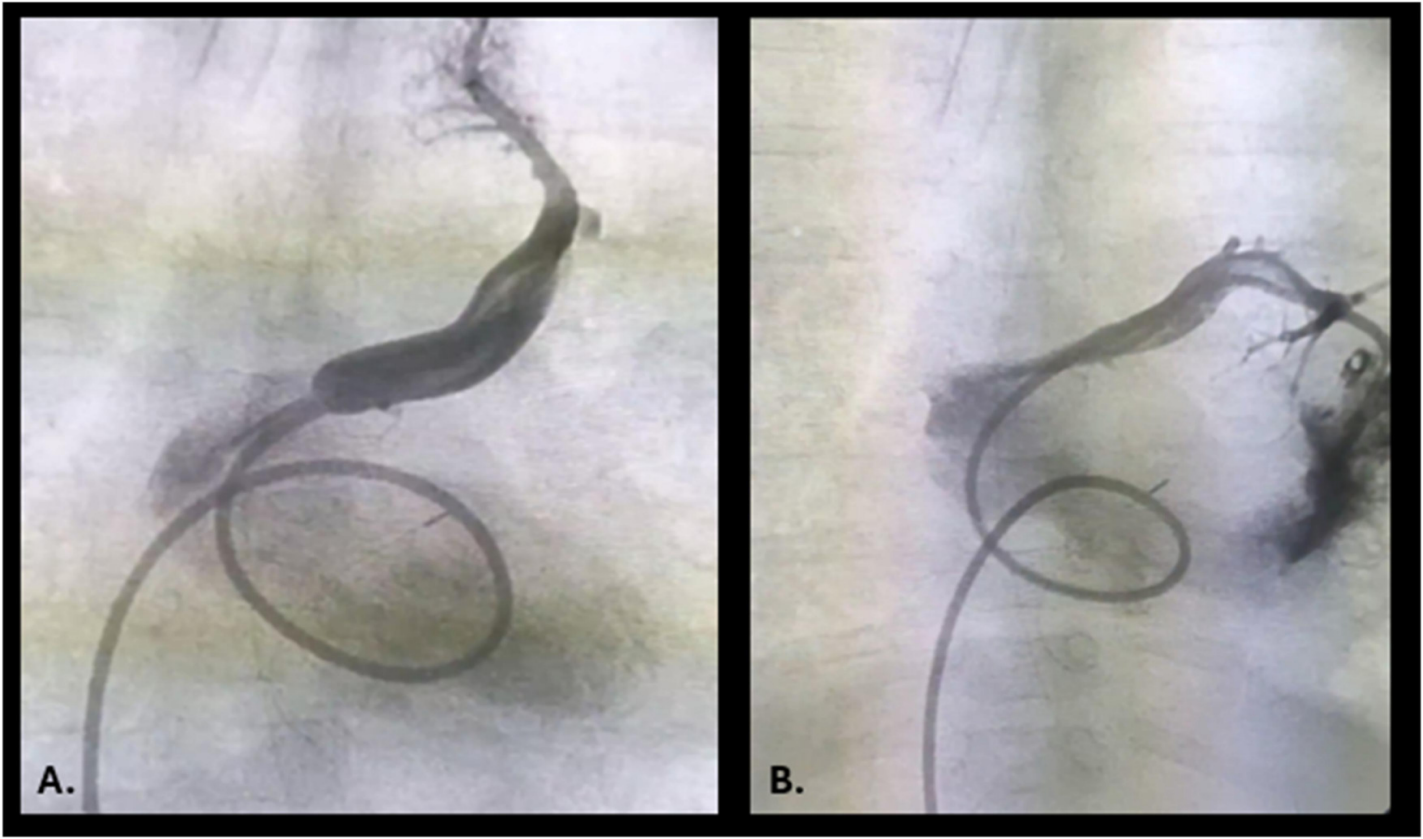
Venography of the left upper pulmonary vein before **(A)** and after **(B)** balloon angioplasty.

At three months of age, he was hospitalised for respiratory distress, feeding intolerance, and early fatigability. Echocardiography examination revealed mild flow acceleration between the pulmonary venous confluence and the left atrium (V max 2 m/s), while the tricuspid PG was 66 mmHg. Cardiac catheterisation pointed out precapillary pulmonary hypertension (PH) with a positive vasoreactivity test using iNO (Table 1). While a decrease in mean pulmonary arterial pressure (mPAP) of 15 mmHg was observed, Ca2 + channel blocker treatment was initiated in addition to previous therapy (bosentan, sildenafil and diuretics). Despite conservative treatment, only mild clinical improvement was observed. However, CT angiography revealed an unobstructive connection between the left atrium and the pulmonary venous confluence (PVC), with compression of both left pulmonary veins by surrounding structures and possible mild stenosis of the left upper pulmonary vein (LUPV). Persistence of PH CT signs was observed. He underwent re-operation using a sutureless repair technique with creation of an atrial septal defect (ASD). The postoperative course was complicated by subtotal LUPV occlusion. Consequently, multiple balloon venoplasties were performed using high-pressure, non-compliant (NC) coronary balloons (Accuforce™ PTCA Dilatation Catheter) at the 7th and 9th months of age, with pressure reduction in LUPV (from 23/17/19 mmHg to 12/8/9 mmHg) (Figure 2). Hemodynamic and oximetric measurements were consistent with mixed PH. Sirolimus, losartan and bosentan therapy were initiated. Systemic infections (Acinetobacter sepsis and influenza infection), acute respiratory distress syndrome and mechanical ventilation dependency complicated the in-hospital course. Control CT angiography in the 1st year of life revealed severe heterogeneous pulmonary parenchymal damage, with subocclusion of the LUPV connecting via collaterals with the left lower pulmonary vein (LLPV). Due to prolonged dependence on invasive mechanical ventilation, tracheostomy was performed. Repeat cardiac catheterisation in the 1st year of life revealed severe mixed, but dominant precapillary PH with complete occlusion of the LUPV (Table 1). He was discharged 4 months after tracheostomy on home mechanical ventilation and full medical treatment. During the two-year follow-up period, repeated polysonographies were done, he was separated from his home ventilator, and decannulation was planned. Before the scheduled procedure, polysomnography, CT angiography, and heart catheterisation were performed. Polysomnography revealed the absence of alveolar hypoventilation, but hypoxiemia, which was corrected with supplemental oxygen therapy. Invasive haemodynamic assessment revealed severe precapillary PH (mean PA pressure 98 mmHg, PVRi 12. 8 Wu/m^2^, wedge 14 mmHg). At the same time, CT angiography showed no pulmonary vein obstruction and the development of an intraparenchymal anastomosis between the LUPV and LLPV. Due to disease progression, the procedure was postponed, and regular oxygen treatment was initiated.

**Table 1:** Report of postoperative echocardiographies, CT scans, and invasive measurements performed simultaneously.

Catheterization				
	No1 (3 months)	No2 (7 months)	No3 (12 months)	No4 (3rd year)
Systemic pressure	80/42/49	75/40/50	78/48/55	90/45/50
SCV (mmHg)	6/2/4			13/11/12
RA (mmHg)	3/0/1			13/7/10
RV (mmHg)	117/0/7	80/8/53	73/0/10	127/0
PA (mmHg)	68/22/38	68/39/50	74/40/55	142/74/98
Wedge (mmHg)	LPA 8–12	LPA 10	LPA 19	14
	RPA 10–16	RPA 11	RPA 18	
TPG (mmHg)	26	40	36	84
LA (mmHg)	9/7/8			11/2/7
PVRi (WU/m2)	5.6	5.2	11.7	12.8
Vasoreactivity test	positive			negative
Qp:Qs	1:1	0.9:1	1:1	0.4:1
Echocardiography				
TV TR (+)	1–1.5	2	1–1.5	1
TV PG (mmHg)	66	90	46	84
PVC (m/s)	2	2.2	1.5	1.5
CT Angiography				
RUPV	5.8		4.7 × 4.2	
RLPV	4.9		4.6 × 4.1	
LUPV	3.7 × 2.5		2.5	Unobtrusive anastomosis with LLPV
LLPV	5.9 × 2.1		4.6 × 3.6	
Parenchyma	septal thickening and consolidation of the pulmonary parenchyma		severe pulmonary parenchymal damage	GGO

SCV, superior caval vein; RA, right atrium; RV, right ventricle; PA, pulmonary artery; LA, left atrium; PVR, pulmonary vascular resistance; LPA, left pulmonary artery; RPA, right pulmonary artery; TV, tricuspid valve; TR, tricuspid regurgitation; PG, pressure gradient; PVC, pulmonary venous confluence; RUPV, right upper pulmonary vein; RLPV, right lower pulmonary vein; LUPV, left upper pulmonary vein; LLPV, left lower pulmonary vein; GGO, ground glass opacity.

**Figure 3:**
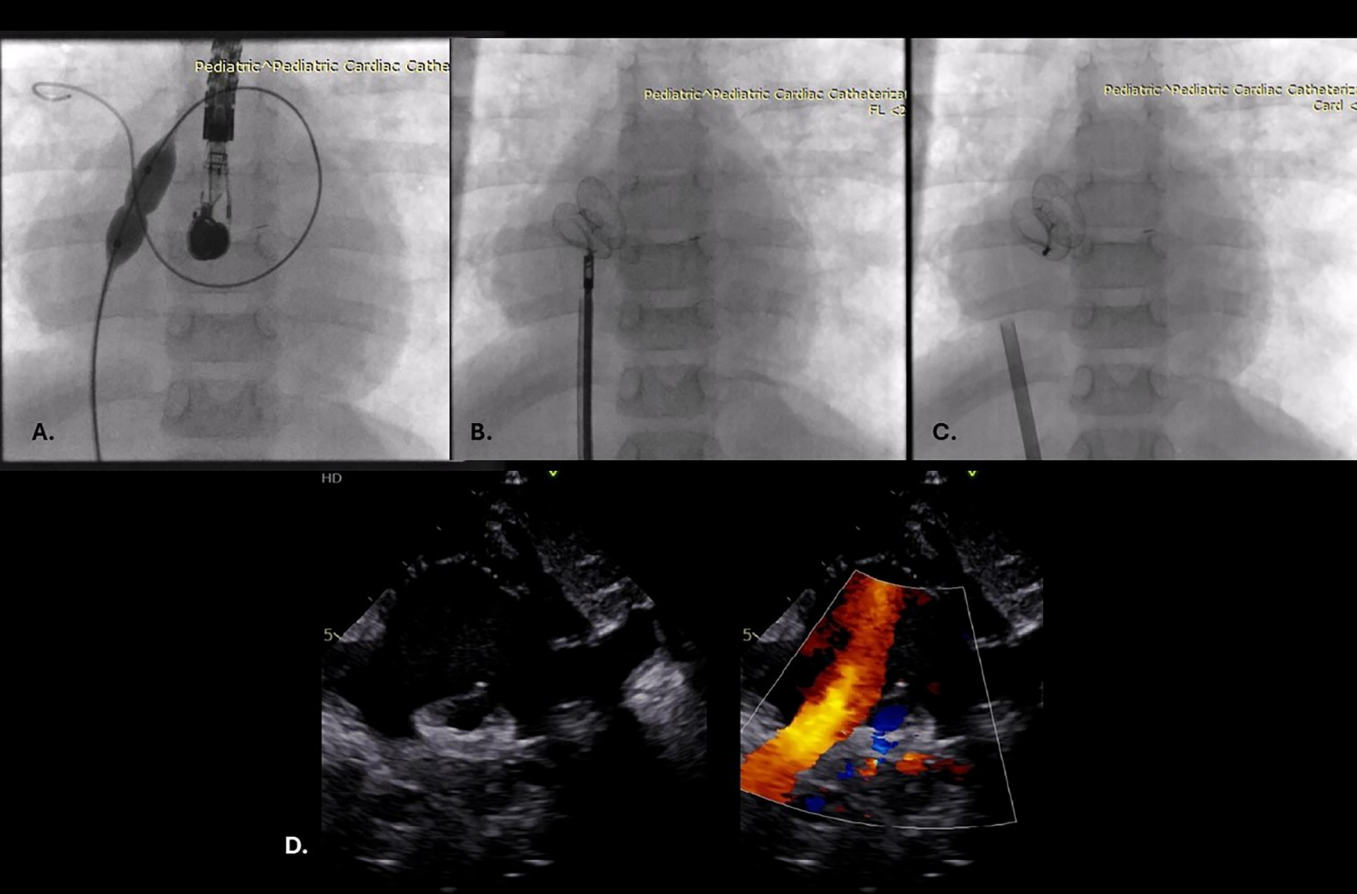
Atrial septal defect predilatation using Z-Med 2 balloon 10 × 20 mm **(A)**; atrial flow regulator (AFR) 8 × 5 mm connected to the delivery cable **(B)**; released AFR **(C)** echocardiographic examination revealed good device position with bidirectional flow **(D)**.

A month after re-evaluation, he was admitted to the ICU due to deterioration of his general condition and progression of PH (BW 10 kg). Echocardiography revealed severe right ventricular dysfunction, severe tricuspid regurgitation, and a restrictive right-to-left shunt on a small ASD (3 mm), while NTproBNP was 44,910 pg/mL. Inhaled NO, along with high-dose milrinone, was initiated; due to unfavourable clinical course, we decided to proceed with implantation of an atrial flow regulator (AFR). The intervention was performed on iNO and milrinone. ASD dilatation was performed using a Z-Med 2 balloon (10 × 20 mm). During balloon insufflation, severe bradycardia with electromechanical dissociation was observed. Adrenaline infusion was initiated, and repeated boluses were administered. Occlutech® AFR 8 × 5 was successfully implanted via 9F delivery sheath (Figure 3). Post AFR implantation, he required ongoing support with noradrenaline, milrinone, dobutamine and levosimendan, with subsequent dose tapering. Control echocardiography demonstrated right ventricular unloading and regression of tricuspid regurgitation, with appropriate device position and presence of right-to-left shunt. Intravenous iloprost treatment was initiated 24 h after the intervention, along with noradrenalin and mirinone. Quick hemodynamic stabilisation was achieved, and inotropes and iloprost were successfully stopped 6 days after intervention. Mechanical ventilation on the home ventilator was initiated, with subsequent conditions being tapered. The patient was discharged with dual antiplatelet therapy, including his heart failure drugs. The cyclic intermittent intravenous iloprost treatment was initiated. During the follow-up period, percutaneous oxygen saturation was around 90%, NTproBNP decreased (last value 1581 pg/mL), while echocardiography revealed a stable PH (PG on TV around 42 mmHg) with a bidirectional interatrial shunt. Systolic RV function was mildly impaired [Tricuspid Annular Plane Systolic Excursion (TAPSE) 13 mm, Tissue Doppler Imaging (TDI) r' 7 cm/s]. The genetic test for PH was negative.

## Discussion

Development of precapillary PH is a rare complication of vascular remodelling and is associated with a poor prognosis. Our patient had several major risk factors, including preoperative pulmonary venous obstruction, preoperative CT signs of PH, an operation on the third day of life with a BW below 3 kg, and postoperative pulmonary venous obstruction. Namely, the first CT angiography revealed PVC as a thin, paravertebral, right-sided vertical vein into which the pulmonary veins drained, with an approximate distance of 20 mm between the upper and lower veins. We are confident that this is the major risk factor for precapillary PH in our case. Regarding obstructive TAPVC, our patient likely developed PH *in utero*. Although prenatal diagnosis is uncommon, it causes severe, immediate postnatal cyanosis and deterioration, necessitating urgent surgical intervention (7). Moreover, our patient exhibited severe pulmonary parenchymal lesions, which contributed to increased PVR. The elevated transpulmonary gradient (TPG) observed in our patient confirmed the presence of pulmonary vascular remodelling, including medial hypertrophy, intimal thickening, and possibly plexiform arterial lesions (8). Although PAWP was slightly elevated and appeared normal on heart catheterisations, the discrepancy between PAWP and left atrial pressure may result from advanced pulmonary vascular disease, heightened capillary hypertension, and pulmonary venous stenosis and occlusion (9, 10). Additionally, chronic congestion and wall shear stress lead to endothelial dysfunction. The pulmonary endothelin pathway is often upregulated, driving further constriction and remodelling, a key mechanism in the transition from mixed to precapillary PH (11).

Due to ongoing LUPV stenosis, balloon venoplasty was performed using coronary balloons alongside simultaneous sirolimus therapy. Postoperative PVS remains a persistent challenge for congenital cardiologists, and the literature indicates that strategies to relieve stenosis, such as reoperation and transcatheter intervention, are limited (4). Sirolimus has recently been reported to be associated with improved survival in paediatric patients with PVS (5). Despite conservative treatment and balloon venoplasty, complete LUPV occlusion was observed in our patient, leading to subsequent intraparenchymal anastomosis to the LLPV, which resolved the elevated pulmonary wedge pressure. Consequently, pulmonary vasodilators were added to therapy, without concern for acute pulmonary edema related to post-capillary PH. Although our patient was on full pulmonary antihypertensive therapy, the clinical condition worsened, prompting consideration of creating a right-to-left shunt to augment systemic output. Namely, it has been observed that primary PH patients with a patent foramen ovale generally have a longer lifespan than those without a shunt. Therefore, targeted interventions were performed in selected PH patients, including standard procedures such as atrial septostomy or surgical approaches such as the Potts shunt and pulmonary artery denervation (12). These techniques improve cardiac function and quality of life while serving as a “bridge to transplant,” often reducing the need for expensive, high-dose intravenous pulmonary vasodilator therapies. In theory, the advantage of a surgical or transcatheter Potts shunt over atrial septostomy is that it avoids upper-body oxygen desaturation, including the brain and coronary arteries. On the other hand, it is associated with higher early postoperative complications (around 25% in some studies) and does not unload the right ventricle because it mainly diverts flow from the pulmonary arterial system to the lower-body systemic arteries (13, 14). Consequently, creation of interatrial communication is an excellent option for patients who cannot tolerate a major surgical procedure. Various techniques and devices have been used to create ASDs, such as atrial septal balloon angioplasty or atrial septal stent implantation, each with its own advantages and disadvantages (15). Balloon septoplasty has low durability, whereas stent implantation is a challenging procedure with a risk of stent protrusion and dislocation. AFR devices have demonstrated good short-term patency and a low complication rate (15). Although the risk of paradoxical embolism exists, we decided to AFR implantation as a life-saving procedure in our patient.

At the age of 3, the patient (BW 10 kg) experienced clinical deterioration in his general condition and progression of PH, so the decision was made to implant an AFR as a life-saving procedure. The promising results of AFR in adults and children with advanced PH were shown by Sivakumar et al. (16). The intent was to improve RV unloading and LV cardiac output. Our patient was resuscitated during the heart catheterisation and balloon septoplasty due to occlusion of ASD as RV pop off, and compromise of LV filling. Additionally, the patient showed significant improvement in clinical condition after AFR implantation. Namely, clinical improvement achieved after AFR implantation in patients with PH results from RV decompression, thereby improving cardiac output and, in turn, end-organ function (17).

Bautista-Rodriguez et al. and Baruteau et al. concluded in their case series that the AFR can be safely deployed in children weighing less than 10 kg and 5 kg through sheaths that are 1–2 Fr smaller than the manufacturer's recommended size (18, 19). We deployed AFR 8 × 5 mm using a 3 size smaller delivery system than recommended (9F delivery system). The patient was resuscitated during the intervention, and after the procedure, cyclic intermittent intravenous iloprost treatment was initiated (20). The patient was successfully weaned from inotropic drug support and returned to home ventilator. The feasibility and clinical impact of AFR implantation via an undersized delivery system in paediatric end-stage PH are promising as a bridge to transplant.

A significant limitation of our manuscript is that it relies on a single patient's experience. Additionally, further investigations should be undertaken to confirm the significance of AFR in pediatric PH and delivery-system downsizing.

This case report highlights the serious risks of ongoing precapillary pulmonary hypertension after surgical correction of obstructive TAPVC. Despite vigorous medical and surgical efforts, our patient progressed to end-stage disease. The successful placement of an AFR 8 × 5 mm through a 9F delivery system- smaller than the usual size- proves its effectiveness as a life-saving measure for unloading the right ventricle and stabilising hemodynamics in a critically ill pediatric patient. This case advocates for broader use of AFR in carefully chosen pediatric patients with advanced PH, even when smaller-than-recommended delivery sheaths are used.

## Data Availability

The original contributions presented in the study are included in the article/supplementary material, further inquiries can be directed to the corresponding author.
